# Hepatic endotheliitis in Golden Syrian hamsters (*Mesocricetus auratus*) experimentally infected with SARS-CoV-2

**DOI:** 10.1590/S1678-9946202466044

**Published:** 2024-07-29

**Authors:** Alex Junior Souza de Souza, Antônio Francisco de Souza, Cristina Kraemer Zimpel, Marina Caçador Ayupe, Marcelo Valdemir de Araújo, Rafael Rahal Guaragna Machado, Erika Salles, Caio Loureiro Salgado, Mariana Silva Tavares, Taiana Tainá Silva-Pereira, Paula Carolina de Souza, Edison Luiz Durigon, Marcos Bryan Heinemann, Paulo Eduardo Brandão, Denise Morais da Fonseca, Ana Marcia de Sá Guimarães, Lilian Rose Marques de Sá

**Affiliations:** 1 Universidade de São Paulo, Faculdade de Medicina Veterinária e Zootecnia, Departamento de Patologia, São Paulo, São Paulo, Brazil; 2 Universidade de São Paulo, Instituto de Ciências Biomédicas, Departamento de Microbiologia, São Paulo, São Paulo, Brazil; 3 Universidade de São Paulo, Faculdade de Medicina Veterinária e Zootecnia, Departamento de Medicina Veterinária Preventiva e Saúde Animal, São Paulo, São Paulo, Brazil; 4 Universidade de São Paulo, Instituto de Ciências Biomédicas, Departamento de Imunologia, São Paulo, São Paulo, Brazil; 5 Instituto Butantan, Centro de Desenvolvimento e Inovação, Laboratório de Virologia, São Paulo, São Paulo, Brazil

**Keywords:** Animal model, COVID-19, Endothelialitis, Hepatitis, Liver

## Abstract

Hepatic injuries in COVID-19 are not yet fully understood and indirect pathways (without viral replication in the liver) have been associated with the activation of vascular mechanisms of liver injury in humans infected with SARS-CoV-2. Golden Syrian hamsters are an effective model for experimental reproduction of moderate and self-limiting lung disease during SARS-CoV-2 infection. As observed in humans, this experimental model reproduces lesions of bronchointerstitial pneumonia and pulmonary vascular lesions, including endotheliitis (attachment of lymphoid cells to the luminal surface of endothelium). Extrapulmonary vascular lesions are well documented in COVID-19, but such extrapulmonary vascular lesions have not yet been described in the Golden Syrian hamster model of SARS-CoV-2 infection. The study aimed to evaluate microscopic liver lesions in Golden Syrian hamsters experimentally infected with SARS-CoV-2. In total, 38 conventional Golden Syrian hamsters, divided into infected group (n=24) and mock-infected group (n=14), were euthanized at 2-, 3-, 4-, 5-, 7-, 14-, and 15-days post infection with SARS-CoV-2. Liver fragments were evaluated by histopathology and immunohistochemical detection of SARS-CoV-2 Spike S2 antigens. The frequencies of portal vein endotheliitis, lobular activity, hepatocellular degeneration, and lobular vascular changes were higher among SARS-CoV-2-infected animals. Spike S2 antigen was not detected in liver. The main results indicate that SARS-CoV-2 infection exacerbated vascular and inflammatory lesions in the liver of hamsters with pre-existing hepatitis of unknown origin. A potential application of this animal model in studies of the pathogenesis and evolution of liver lesions associated with SARS-CoV-2 infection still needs further evaluation.

## INTRODUCTION

Hepatic “endotheliitis” was originally applied to describe the attachment of lymphoid cells to the luminous surface of endothelial cells of portal vessels and/or hepatic terminal veins^1^. Currently, it is known that hepatic endotheliitis represents an important predictive factor for acute liver allograft rejection in humans, combined with portal inflammation and bile duct damage^2-6^.

Hepatic endothelial activation and endotheliitis may also be associated with viral and autoimmune hepatitis, primary biliary cholangitis, nonalcoholic steatohepatitis, drug-induced or chronic hepatitis in humans^2,4,6^; and in mice it is used as a graft versus host disease (GVHD) model^5^.

The microscopic lung lesions of COVID-19 in humans are characterized by diffuse alveolar damage, progressive bronchopneumonia, and vascular events including endotheliitis, peri/vasculitis, hemorrhage, microthrombosis, and intravascular hemolysis^7-9^. The pulmonary and/or systemic vascular lesions in acute phase of COVID-19 are usually related to the presence of SARS-CoV-2 in the lung microenvironment and are associated with an exacerbated systemic immune response to the infection^7-9^.

The occurrence of vascular, degenerative, and/or inflammatory lesions in the liver of humans with COVID-19 is not yet fully understood, but an increasing number of cholangiopathies and hepatic diseases associated with SARS-CoV-2 infection have been identified^9-14^. Although human hepatocytes express angiotensin-converting enzyme 2 (ACE2)—the main receptor used for virus entry into cells—the existence of liver damage due to SARS-CoV-2 replication in the liver is still controversial^11-13^.

Factors such as SARS-CoV-2 variants, hypoxia and ischemia-reperfusion injuries, drug-induced damage, and concomitant liver diseases may be related to varying degrees of liver disorders observed in humans with COVID-19^11^. Systemic inflammation resulting from cytokine storm and extrahepatic vascular damage have also been proposed as factors that may contribute to liver injury in COVID-19^8-14^. Additionally, coagulation mechanisms and sinusoidal endothelial damage associated with hepatic disorders in SARS-CoV-2 infection in humans can be indirectly activated (without evidence of viral replication in the liver), for example by the IL-6 trans-signaling pathway^12^.

Golden Syrian hamsters (*Mesocricetus auratus*) are used as an effective experimental model for reproducing acute, moderate, self-limiting lung disease during SARS-CoV-2 infection^15-18^. In this experimental model—even without evidence of viral replication in lung endothelial cells—intranasal SARS-CoV-2 infection activates a potent pro-inflammatory and antiviral response that induces the recruitment and adhesion of T lymphocytes in the intima of blood vessels that causes endotheliitis lung lesions^17^.

The occurrence of extrapulmonary vascular lesions (including endotheliitis) has not yet been demonstrated in this experimental model of SARS-CoV-2 infection^16^. Regarding hepatic lesions in Golden Syrian hamsters infected with SARS-CoV-2, there is only one description of random hepatic necrosis with rare inflammatory infiltrate, and cholecystitis in animals intranasally inoculated with a high dose SARS-CoV-2^15^.

This study aimed to evaluate microscopic liver lesions in Golden Syrian hamsters experimentally infected with SARS-CoV-2 in two independent experiments of unrelated studies.

## MATERIALS AND METHODS

The experimental procedures were approved by the Institutional Animal Care and Use Committee, Institute of Biomedical Sciences, University of Sao Paulo (protocols Nº 2530190221 and 5167261020).

The study included 38 (n = 38) male, 3-month-old, conventional hamsters, divided into two groups: 24 animals (infected group, IG) were intranasally inoculated with 10^5^ 50% tissue culture infective dose (TCID_50_) of SARS-CoV-2 strain B.1.1.28 (GenBank accession Nº MT126808.1), in 50 μL of vehicle Dulbecco’s Modified Eagle Medium (DMEM) with 10% fetal bovine serum (FBS), and were euthanized in 2-(n=3), 3- (n=3), 4-(n=3), 5-(n=3), 7-(n=6), 14-(n=6) days post-inoculation (dpi); and 14 animals (mock-infected group, MG) intranasally inoculated with 50 μL DMEM with 10% FBS that were euthanized at 2- (n=3), 4-(n=2), 7-(n=3), 14-(n=2), and 15-(n=4) dpi. Noteworthy, conventional hamsters were used because specific pathogen-free hamsters are unavailable in Brazil.

Lung and liver fragments fixed in 10% buffered formaldehyde solution were processed for histopathology and immunohistochemical (IHC) detection of Spike S2 [mAb anti-coronavirus (SARS-CoV-2) spike S2, mouse, 1:300, catalog Nº 08720402 MP Biomedicals], used in combination with a polymer detection system (REVEAL^®^ Polyvalent HRP-DAB Detection System, Spring Bioscience) and a red chromogen (ImmPACT NovaRED^TM^ chromogen, Vector Laboratories).

The intensity and distribution of portal and interface hepatitis, confluent necrosis, lobular activity (focal lytic necrosis, apoptosis, and focal inflammation) and fibrosis were semi-quantified based on the Ishak score system^3^. Table 1 describes additional parameters.

**Table 1:** Parameters of degenerative and vascular microscopic liver lesions in Golden Syrian hamsters (*Mesocricetus auratus*).

Histological parameter	Type of lesion	Intensity^a^
Hepatocellular degeneration	0 = absent, 1 = ballooning 2 = steatosis	0 = absent 1 = <33% 2 = >33% to <66% 3 = >66%
Portal vascular lesion	0 = absent 1 = congestion 2 = endothelial swelling 3 = endotheliitis 4 = vasculitis	0 = absent 1 = <33% 2 = >33% to <66% 3 = >66%
Lobular vascular lesion	0 = absent 1 = congestion 2 = hemorrhage 3 = sinusoidal dilatation	0 = absent 1 = <33% 2 = >33% to <66% 3 = >66%
^a^According to the occurrence/distribution in the liver microscopic area.		

The intensity of endotheliitis was evaluated using the adapted Birmingham System^19^, considering: 0 = no portal tracts involved; 1 = focal attachment of lymphoid cells to endothelial surface with no prominent subendothelial infiltration; 2 = more extensive lymphoid attachment with conspicuous subendothelial infiltration; 3 = extensive subendothelial infiltration with lifting and prominent focal disruption. We also performed Aquaporin-1 (AQP1) immunostaining on histological sections of IG and MG animals to reinforce the morphological diagnosis of endotheliitis in the hamsters’ lung and liver (Supplementary Figure S1).

## RESULTS

The IG presented a moderate, self-limiting respiratory disease with weight loss (~10%) and general condition recovery within 14 days, like other studies^15-18^. Lung samples were subjected to virus isolation and RT-qPCR (data not shown) that confirmed viral replication in the respiratory tract and the development of acute respiratory disease only in IG.

Microscopic lesions in the lung parenchyma were bronchiolitis/peribronchiolitis, with progression to interstitial bronchopneumonia. There were IHC detection of Spike S2 antigen in bronchial/bronchiolar epithelium and type I pneumocytes between 2 and 5 dpi. After the fifth day, there was a pulmonary regenerative response characterized by dysplastic type II pneumocyte hyperplasia, and the resolution of the acute disease occurred between 7 and 14 dpi, as previously described^15-18^. Photomicrographs of lung histopathology and immunohistochemistry in the SARS-CoV-2 experimental infection in Golden Syrian hamsters are available in Supplementary Figure S2.

**Figure 1:**
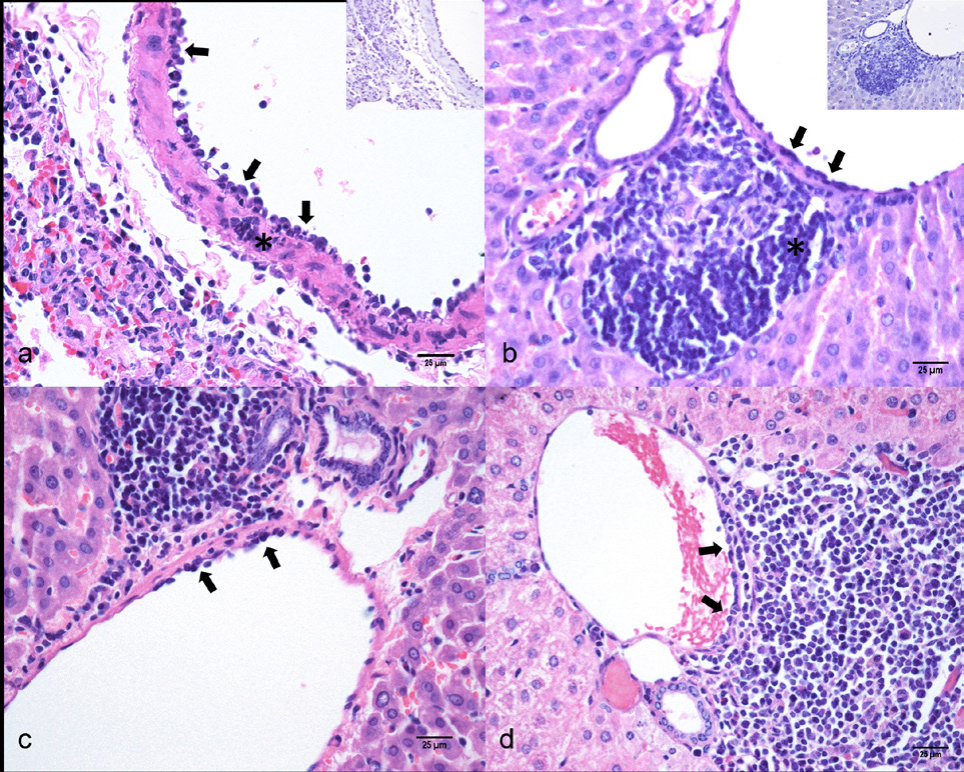
Microscopic lesions of SARS-CoV-2 experimental infection in Golden Syrian hamster (*Mesocricetus auratus*): a) Lung, 4-days post-inoculation (dpi), Infected-group (IG), endotheliitis (arrows) and vasculitis (*). Hematoxylin and eosin (HE), bar 25 µm. Inset: Lung, 2-dpi, positive Spike S2 immunostaining on bronchiolar epithelium and negative on vascular endothelium and lymphocytes; b) Liver, 2-dpi, IG, portal endotheliitis (arrows) and portal hepatitis (*). HE, bar 25 µm. Inset: negative Spike S2 immunostaining on vascular endothelium, inflammatory cells and adjacent liver tissue; c) Liver, 2-dpi, IG, discrete (score 1) portal vein endotheliitis (arrows), characterized by a small number of lymphocytes adhered to the luminal surface of endothelial cells, HE, bar 25 µm; d) Liver, 14-dpi, moderate (score 2) portal vein endotheliitis (arrows), IG, more extensive lymphoid attachment with prominent subendothelial infiltration, HE, bar 25 µm.

Between 2 and 5 dpi, pulmonary vascular changes were characterized by hemorrhage, endothelial swelling, endotheliitis (Figure 1a), vasculitis, and perivasculitis, without spike S2 immunodetection in vascular endothelium and/or inflammatory cells in the vessel wall (Figure 1a). Up to 14 dpi, mild multifocal lymphocytic perivasculitis was still detected.

For hepatic histopathological analysis, we evaluated three to four liver fragments (mean±SD; 3.49±0.37), containing a mean of 52 portal spaces (range±SD; 23-85±15.89) per animal. Mild to moderate lymphohistiocytic portal hepatitis was detected in 83% (20/24) of the hamsters within IG and 78.57% (11/14) of the hamsters in the MG (Supplementary Figure S3), with lymphoid portal aggregates/follicles observed in 58.33% (14/24) and 78.57% (11/14) of the IG and MG, respectively. Discrete interface hepatitis was observed in 58.33% (14/24) of the IG and 57.14% (8/14) of MG.

We observed portal vein endotheliitis (Figure 1b) in 50% (12/24) of the IG and in 28.57% (4/14) of MG animals, an increase of 21.43% in frequency of affected animals between MG and IG (Supplementary Figure S1). This lesion was characterized by a small number of lymphocytes adhered to the luminal surface of endothelial cells and/or beneath the endothelium, with focal to multifocal distribution. In the IG, the endotheliitis lesion was observed at 2-(3/3), 3-(2/3), 4-(1/3), 5-(1/3), 7-(2/6), 14-(3/6) dpi; and in MG the lesion was observed in 2-(2/3), 7-(1/3), 14-(1/2) dpi.

In the IG, eight hamsters presented score 1 (Figure 1c) and four presented score 2 of endotheliitis (Figure 1d); while among MG, two animals were scored as 1 and two scored 2. At all experimental times and in both groups, S2 IHC detection was negative in vascular endothelium, biliary epithelium, and/or adjacent liver parenchyma (Figure 1b).

In addition to portal endotheliitis, the frequency of lobular vascular changes was also higher in the IG, 29.16% (7/24), compared to the MG, 14.29% (2/14). Within the IG, six cases presented mild sinusoidal congestion and one showed sinusoidal dilatation; MG-affected animals presented sinusoidal congestion.

The frequency of mild focal to multifocal hepatocellular degeneration (ballooning or microvesicular steatosis) and lobular activity were also higher in the IG compared to the MG. Accordingly, mild focal to multifocal hepatocellular degeneration was observed in 87.50% (21/24) of the IG and 71.42% (10/14) of the MG; and mild to moderate lobular activity (infiltrate composed of lymphocytes, histiocytes, and few heterophils) in 79.16% (19/24) of the IG and 64.28% (9/14) in the MG. Table 2 shows the frequencies of liver lesions.

**Table 2:** Frequency distribution of microscopic liver lesions in Golden Syrian hamsters (*Mesocricetus auratus*) in SARS-CoV-2 infected group (IG) and mock-infected group (MG).

Hepatic lesions	Days post infection (dpi)										

	2		3	4		5	7		14		15

	IG	MG	IG	IG	MG	IG	IG	MG	IG	MG	MG
Endotheliitis	3/3	2/3	2/3	1/3	0/2	1/3	2/6	1/3	3/6	1/2	0/4
Portal hepatitis	3/3	2/3	2/3	3/3	2/2	2/3	5/6	3/3	5/6	2/2	2/4
Interface hepatitis	3/3	2/3	1/3	2/3	2/2	1/3	5/6	3/3	2/6	1/2	0/4
Lobular activity	3/3	3/3	3/3	3/3	2/2	1/3	5/6	3/3	4/6	1/2	0/4
Hepatocellular degeneration	3/3	1/3	3/3	2/3	2/2	3/3	4/6	1/3	6/6	2/2	4/4
Lobular vascular changes	0/3	0/3	1/3	2/3	2/2	2/3	2/6	0/3	0/6	0/2	0/4

## DISCUSSION

The original morphological criteria for the diagnosis of endotheliitis were established by the observation of at least one lymphoid cell adhered to the lumen of the endothelium and/or beneath it^1,4-6^. Additionally, the severity/intensity of endotheliitis ranges from a focal/segmental fixation of single cells or groups of lymphoid cells (predominantly T lymphocytes) on the luminal surface of vascular endothelium to subendothelial infiltration of isolated or several lymphocytes^2,4-7^.

In contrast to reports in humans^7-9^, pulmonary endotheliitis in hamsters is not directly related to the detection of SARS-CoV-2 in endothelial cells^16-18^. It has been shown that even in the absence of SARS-CoV-2 viral particles in pulmonary vascular endothelium in hamsters, an intense antiviral and pro-inflammatory transcriptional response of pulmonary endothelial cells may activate chemotaxis molecules that are involved in the attraction of T cells to the vessels^17,18^, which may partially explain the pulmonary endotheliitis response reported here.

The frequency of animals with hepatitis-associated lesions was similar in IG and MG, and hepatitis was a pre-existent condition that was observed in different intensity and distribution. The hepatic endotheliitis in our experiments stands out because other studies in hamsters point to the absence of extra respiratory vascular lesions^16^. In contrast, few studies have reported the occurrence of portal vein endotheliitis with variable degrees in post-mortem liver samples from humans with SARS-CoV-2 infection^9,10,14^. While the hamsters were apparently healthy and are regularly tested for known rodent pathogens, very little is known about causes of pre-existing hepatitis. We believe that SARS-CoV-2 may have exacerbated vascular lesions of previously affected animals. Pre-existing liver conditions may also explain findings of vascular, degenerative, and/or inflammatory lesions in the liver of humans with COVID-19^9-14^.

Considering that hamsters from conventional breeding were used, the existence of comorbidities and/or unknown factors exacerbated by or potentiating SARS-CoV-2-induced hepatic endotheliitis cannot be ruled out and should be investigated. Hamsters are one of the least studied laboratory animal species and many of their metabolic and infectious diseases remain unidentified^20^.

Despite the observation of endotheliitis in IG and MG hamsters without statistical difference (data not shown), we consider that the description of this type of lesion in humans and in animal models of COVID-19 still deserves our highlight and further evaluation by other researchers. For example, in the original study, hepatic endotheliitis was also described in GVHD-negative individuals and there was no statistical difference between groups with and without GVHD (8/24 and 3/72 cases, respectively)^1^. And as in their results, limited number of cases analyses may have influenced the statistical analyses.

The spike S2 antigen was not detected by IHC in the hepatic vascular lesions observed herein, despite the ACE2 expression in liver endothelial sinusoids of Golden Syrian hamsters^15-16^. However, it has been proposed that a systemic pathway of IL-6 trans-signaling may be involved in a mechanism of activation in liver sinusoidal endothelial cells, clotting factors, and platelets in humans infected with SARS-CoV-2^12,13^. In turn, this may be associated with sinusoidal endotheliopathy and activation of potential thromboinflammatory mechanisms related to liver injury in COVID-19^12,13^. We emphasize that the liver lesions described in hamsters differ morphologically from sinusoidal endotheliopathy and activation of thromboinflammatory mechanisms in humans with COVID-19^12,13^. Nevertheless, the observed hepatic portal vein endotheliitis may suggest the existence of indirect mechanisms of venous endothelial activation associated with the viremia stage in the hamster model of COVID-19, which remains to be evaluated.

Additionally, we consider that the sampled liver lobes, the size of fragments (directly related to number of microscopical fields), the number of portal tracts, and/or the depth of histological sections examined may explain, at least partially, the few reports published of vascular and/or inflammatory hepatic lesions in hamsters experimentally inoculated with SARS-CoV-2. In humans, the use of 10 or more consecutive histological sections can improve the microscopic hepatic evaluation during routine liver biopsy analysis^3^. In addition, portal endotheliitis was reported by a study that evaluated two wedge liver fragments containing at least 20 portal spaces in each case of COVID-19^14^. Therefore, we suggest that liver fragments containing at least 10 portal spaces be used for histopathological evaluation in hamsters and other animal models of COVID-19.

## CONCLUSION

Although this report is limited by the lack of pathogenic mechanism for hepatic endotheliitis, our findings suggest a potential application of hamsters in studies of the pathogenesis and evolution of liver injury in SARS-CoV-2 infection and the comparative pathology, which have been hitherto overlooked.
