# Supplementary material for: Hepatic endotheliitis in Golden Syrian hamsters (Mesocricetus auratus) experimentally infected with SARS-CoV-2
Source: Rev Inst Med Trop Sao Paulo. 2024 Jul 29;66:e44. doi: 10.1590/S1678-9946202466044 (PMC11295288; doi:10.1590/S1678-9946202466044)
Supplement: Supplementary file 1 [file 1678-9946-rimtsp-66-S1678-9946202466044-s1.pdf]

Supplementary Material of the article "Hepatic endotheliitis in Golden Syrian hamsters (*Mesocricetus auratus*) experimentally infected with SARS-CoV-2"

Corresponding author: Lilian Rose Marques de Sá
Universidade de São Paulo, Faculdade de Medicina Veterinária e Zootecnia, Departamento de Patologia, São Paulo, São Paulo, Brazil
E-mail: liliansa@usp.br

Data collection date:

Data was collected from 2022 to 2023.

Description of data:

Supplementary files provide additional histopathology and immunohistochemistry (IHC) photomicrographs of Golden Syrian Hamsters (*Mesocricetus auratus*) experimentally infected with SARS-CoV-2. The figures illustrate and reinforce microscopic diagnoses of lung and liver lesions in the animals. For microscopic evaluation, lung and liver fragments fixed in 10% buffered formaldehyde solution were processed to obtain 5 µm histological sections stained with hematoxylin-eosin. The tissues were evaluated under optical microscopy (Eclipse NiU, Nikon, Japan), at magnifications of 4x, 10x, 40x and 100x, and the lesions were photodocumented with a DS–U3 digital camera and Ni elements software (Nikon, Japan). We used anti-Spike S2 antibody [mAb anti-coronavirus (SARS-CoV-2) spike S2, mouse, 1:300, catalog number 08720402 MP Biomedicals] for immunohistochemical detection of SARS-CoV-2 in tissues, and Aquaporin 1 polyclonal antibody, rabbit (PA5-78806, 1:1000, Thermo Fisher Scientifics) for aquaporin 1 immunostaining. In both protocols, a polymer detection system (REVEAL® Polyvalent HRP-DAB Detection System, Spring Bioscience) and a red chromogen (Immpact NovaRed™ chromogen, Vector Laboratories) were used.
