# Supplementary material for: Hepatic endotheliitis in Golden Syrian hamsters (Mesocricetus auratus) experimentally infected with SARS-CoV-2
Source: Rev Inst Med Trop Sao Paulo. 2024 Jul 29;66:e44. doi: 10.1590/S1678-9946202466044 (PMC11295288; doi:10.1590/S1678-9946202466044)
Supplement: Supplementary file 2 [file 1678-9946-rimtsp-66-S1678-9946202466044-s2.pdf]

REVISTA DO INSTITUTO DE MEDICINA TROPICAL DE SÃO PAULO

JOURNAL OF THE SÃO PAULO INSTITUTE OF TROPICAL MEDICINE

SUPPLEMENTARY MATERIAL

https://doi.org/10.48331/scielodata.W35IBM

Hepatic endotheliitis in Golden Syrian hamsters (*Mesocricetus auratus*) experimentally infected with SARS-CoV-2

Alex Junior Souza de Souza[1], **Antônio Francisco de Souza Filho**[2], **Cristina Kraemer Zimpel**[2,3], **Marina Caçador Ayupe**[4], **Marcelo Valdemir de Araújo**[2,4,5], **Rafael Rahal Guaragna Machado**[2], **Erika Salles**[4], **Caio Loureiro Salgado**[4], **Mariana Silva Tavares**[2], **Taiana Tainá Silva-Pereira**[2], **Paula Carolina de Souza**[4], **Edison Luiz Durigon**[2], **Marcos Bryan Heinemann**[3], **Paulo Eduardo Brandão**[3], **Denise Morais da Fonseca**[4], **Ana Marcia de Sá Guimarães**[2], **Lilian Rose Marques de Sá**[1]

[1]Universidade de São Paulo, Faculdade de Medicina Veterinária e Zootecnia, Departamento de Patologia, São Paulo, São Paulo, Brazil

[2]Universidade de São Paulo, Instituto de Ciências Biomédicas, Departamento de Microbiologia, São Paulo, São Paulo, Brazil

[3]Universidade de São Paulo, Faculdade de Medicina Veterinária e Zootecnia, Departamento de Medicina Veterinária Preventiva e Saúde Animal, São Paulo, São Paulo, Brazil

[4]Universidade de São Paulo, Instituto de Ciências Biomédicas, Departamento de Imunologia, São Paulo, São Paulo, Brazil

[5]Instituto Butantan, Centro de Desenvolvimento e Inovação, Laboratório de Virologia, São Paulo, São Paulo, Brazil

Correspondence to: Lilian Rose Marques de Sá
Universidade de São Paulo, Faculdade de Medicina Veterinária e Zootecnia, Departamento de Patologia, Av. Prof. Dr. Orlando Marques de Paiva 87, Cidade Universitária, 05508-270, São Paulo, SP, Brazil

E-mail: liliansa@usp.br

Received: 5 March 2024

Accepted: 3 June 2024

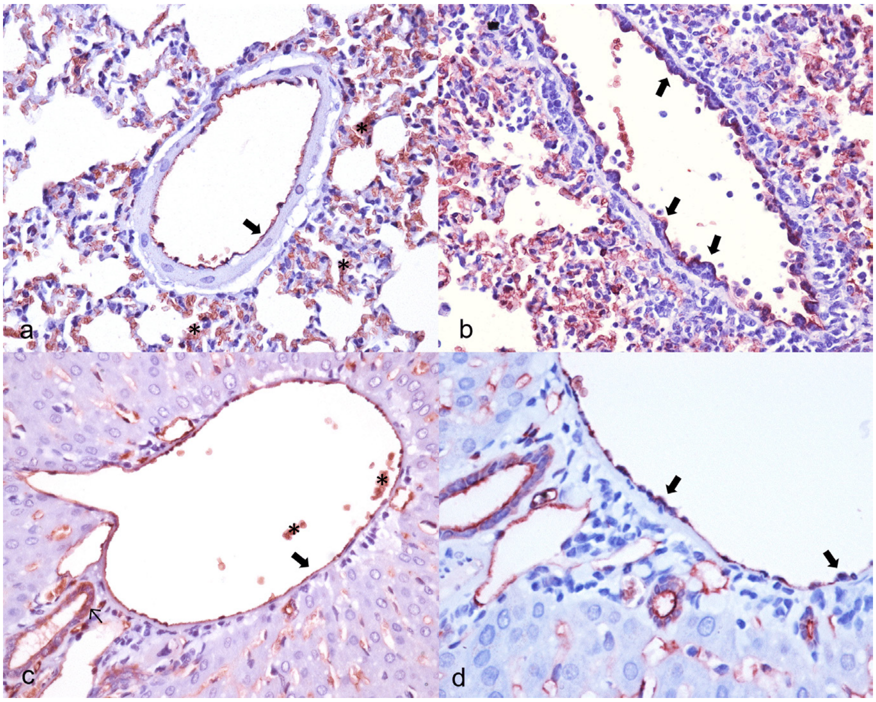

Supplementary Figure S1 - Immunohistochemical detection of aquaporin-1 (AQP1) in Golden Syrian hamster (*Mesocricetus auratus*): a) Lung, mock-infected animal (MG), 2-days post-inoculation (dpi). AQP1 expressed in red blood cells (*) and normal vascular endothelium (arrows). ImmPACT NovaRED™ chromogen, Harris hematoxylin counterstain, 40x; b) Lung, SARS-CoV-2 infected animal (IG), 4-dpi, AQP1 expressed on red blood cells and vascular endothelium showing moderate endotheliitis (arrows). ImmPACT NovaRED™ chromogen, Harris hematoxylin counterstain, 40x.; c) Liver, MG, 4-dpi, AQP1 expressed in red blood cells (*) and normal vascular endothelium (arrows), hepatic sinusoids and cholangiocytes (thin arrow). ImmPACT NovaRED™ chromogen, Harris hematoxylin counterstain, 40x.; d) Liver, IG, 4-dpi, AQP1 expressed in red blood cells, vascular endothelium, hepatic sinusoids, cholangiocytes, and hepatic portal vein showing mild endotheliitis (arrows). ImmPACT NovaRED™ chromogen, Harris hematoxylin counterstain, 60x.

This is an open-access article distributed under the terms of the Creative Commons Attribution License.

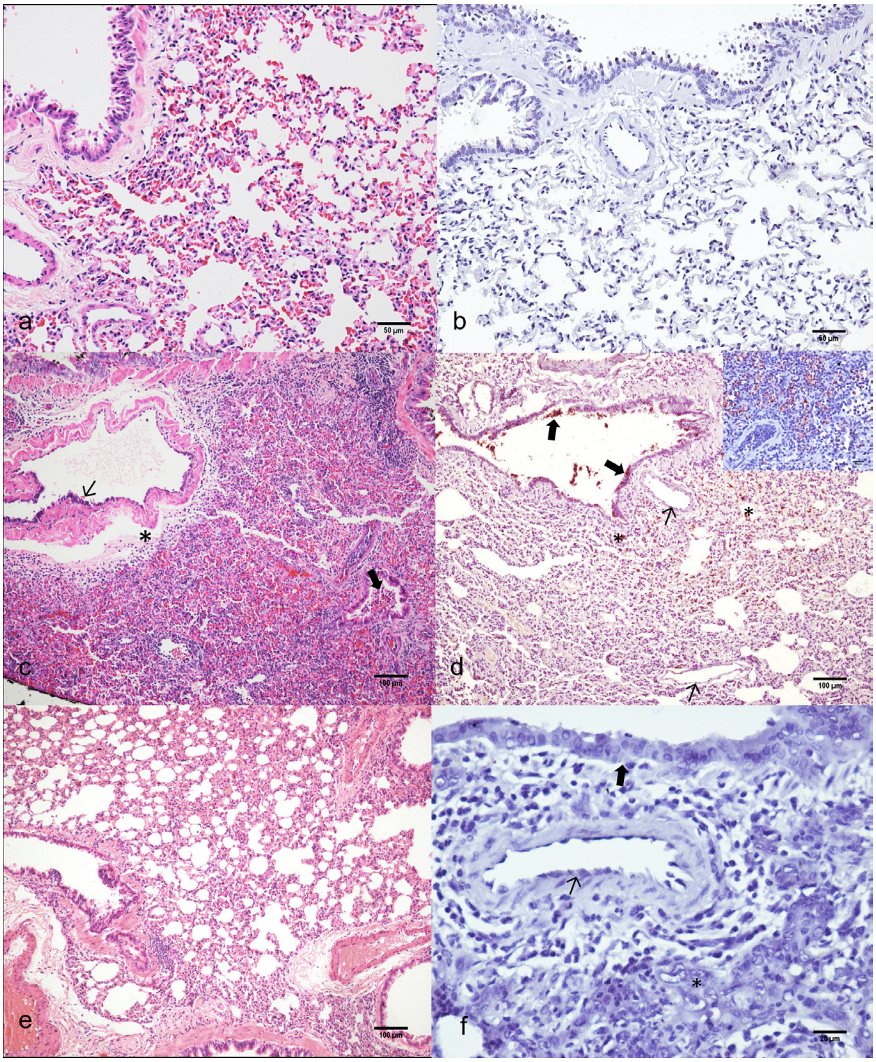

Supplementary Figure S2 - Histopathological and immunohistochemical examination of lung tissues of Golden Syrian hamster (*Mesocricetus auratus*) used in SARS-CoV-2 experimental infection: a) Lung, 2-days post-inoculation (dpi), mock-infected group (MG), histopathology, lung tissue with preserved morphology. Hematoxylin and eosin (HE), 20x; b) Lung, 2 dpi, MG, immunohistochemistry (IHC) anti-coronavirus SARS-CoV-2 spike S2 antigens. Negative immunostaining in lung tissue. ImmPACT NovaRED™ chromogen, Harris hematoxylin counterstain, 20x; c) Lung, 4 dpi, infected group (IG), histopathology, bronchointerstitial pneumonia exhibiting exudate in the bronchiolar lumen (arrow), abundant inflammatory infiltrate in alveolar spaces, perivascular edema (*) and endotheliitis (thin arrow). HE, 10X; d) Lung, 4 dpi, IG, IHC anti-coronavirus SARS-CoV-2 spike S2 antigens, positive immunostaining in ciliated cells (arrows), pneumocytes and alveolar inflammatory cells (*), and negative immunostaining in the endothelium of blood vessels (thin arrows). Inset: Lung, 4-dpi, positive Spike S2 immunostaining on pneumocytes and alveolar inflammatory cells. ImmPACT NovaRED™ chromogen, Harris hematoxylin counterstain, 10x; e) Lung, 14 dpi, IG, histopathology, organizing bronchointerstitial pneumonia. Note the less thickened alveolar septa and less inflammatory infiltrate in the alveolar spaces. HE, 10x; f) Lung, 14 dpi, IG, IHC anti-coronavirus SARS-CoV-2 spike S2 antigens, negative immunostaining in bronchiolar cilliated cells (arrow), blood vessel endothelium (thin arrow) and dysplastic and hyperplastic type II pneumocytes (*). ImmPACT NovaRED™ chromogen, Harris hematoxylin counterstain, 40x.

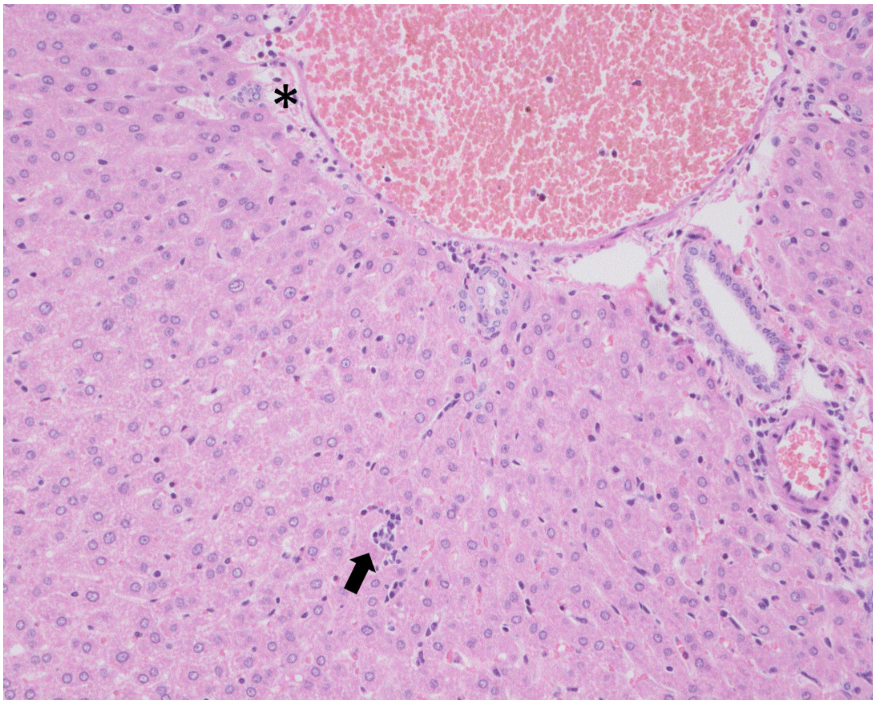

Supplementary Figure S3 - Liver histopathology, 4-days post-inoculation, mock-infected group, minimal lobular (arrow) and portal space (*) inflammatory infiltrate. Hematoxilin and eosin, 20x.
